# Myocardial involvement in desmin myopathy due to a DES c.1360C > T (p.Arg454Trp) variant

**DOI:** 10.1093/ehjcr/ytag400

**Published:** 2026-05-29

**Authors:** Ying Wang, Kai Yang, Minjie Lu

**Affiliations:** Department of Magnetic Resonance Imaging, Fuwai Hospital and National Center for Cardiovascular Diseases, Chinese Academy of Medical Sciences and Peking Union Medical College, Beilishi Road No. 167, Xicheng District, Beijing 100037, China; Department of Medical Imaging, Sanmenxia Central Hospital, Xiaoshan Middle Road, Sanmenxia, Henan 472000, China; Department of Magnetic Resonance Imaging, Fuwai Hospital and National Center for Cardiovascular Diseases, Chinese Academy of Medical Sciences and Peking Union Medical College, Beilishi Road No. 167, Xicheng District, Beijing 100037, China; Department of Magnetic Resonance Imaging, Fuwai Hospital and National Center for Cardiovascular Diseases, Chinese Academy of Medical Sciences and Peking Union Medical College, Beilishi Road No. 167, Xicheng District, Beijing 100037, China

A 14-year-old male patient presented with paroxysmal syncope for more than ten days, occurring exclusively during walking.

Laboratory investigations showed mildly elevated levels of creatine kinase isoenzyme (CK-MB) and lactate dehydrogenase (LDH), as well as significantly elevated levels of B-type natriuretic peptide (BNP) and NT-proBNP. Electrocardiography demonstrated ectopic rhythms, atrial fibrillation, intraventricular conduction block, and ST–T segment abnormalities. Echocardiography revealed enlargement of both atria, thickening of the left ventricular inferolateral wall, and impaired left ventricular diastolic function. Cardiac magnetic resonance imaging (MRI) demonstrated marked biatrial enlargement (left atrial volume index [LAVi], 98.01 mL/m^2^; right atrial volume index [RAVi], 64.02 mL/m^2^; the area of right atrium, 31.51 cm^2^; *Figure 1A*), a left ventricular ejection fraction of 60%, restrictive filling pattern, and left ventricular wall thickening with elevated T1 values (1239 ms; *Figure 1B and 1C*), elevated extracellular volume (22%). Late gadolinium enhancement was observed in the subendocardial layers of the anterior, lateral, and apical walls of the left ventricle (*Figure 1D and 1E*), consistent overall with restrictive cardiomyopathy. MRI of both lower limbs showed reduced muscle volume in the examined muscle groups, accompanied by fatty infiltration and atrophy (*Figure 1F*).

**Figure 1:**
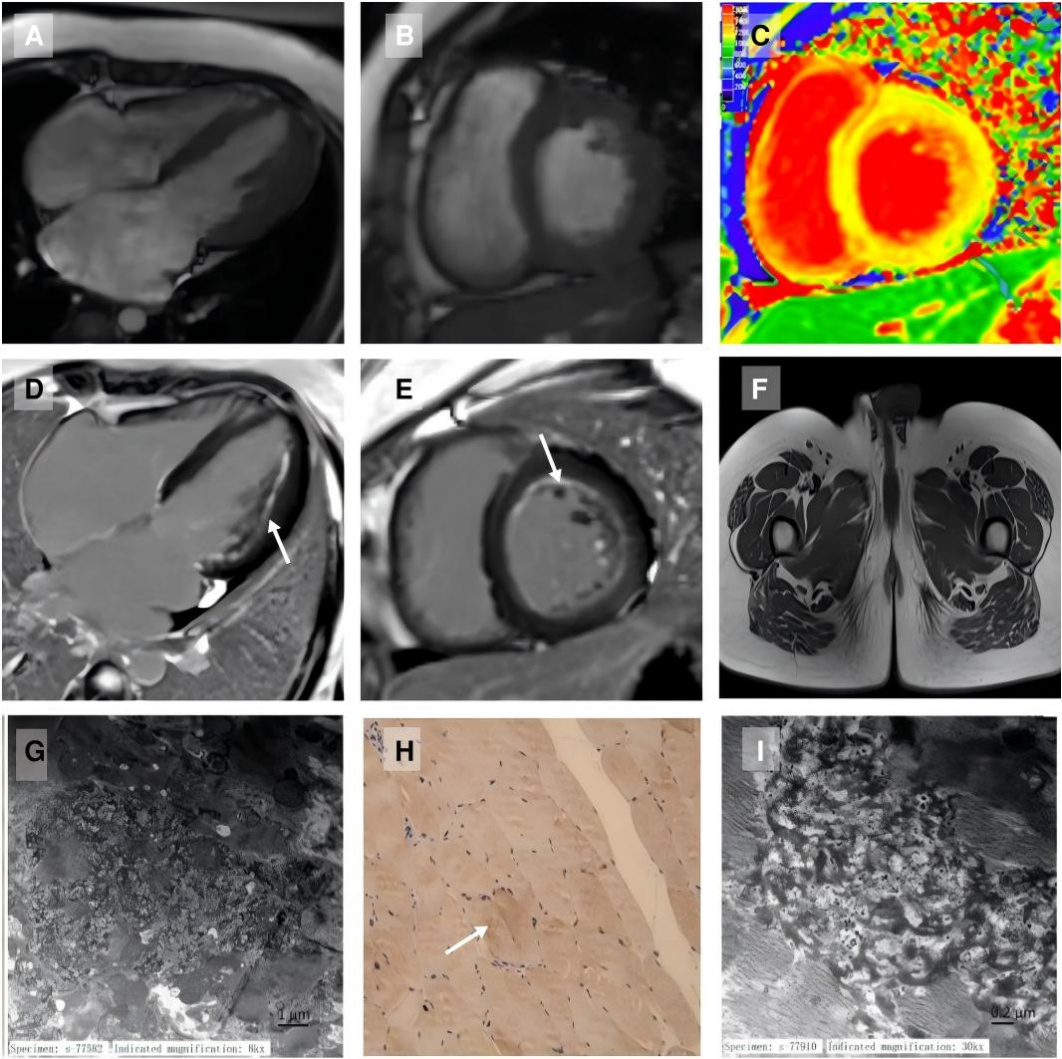
(*A*) Still image of four-chamber still magnetic resonance imaging showing marked biatrial enlargement with relatively normal-sized ventricles. (*B*) Still image of short-axis cine magnetic resonance imaging demonstrating predominant thickening of the left ventricular inferolateral wall (maximum thickness, 14–15 mm). (*C*) Short-axis T1 mapping showing thickened left ventricular myocardium with elevated native T1 values (1239 ms). (*D–E*) Late gadolinium enhancement images demonstrating subendocardial enhancement of the anterior wall, lateral wall, and apex of the left ventricle (white arrows). (*F*): Magnetic resonance imaging of both lower limbs showing reduced muscle volume in the examined muscle groups, accompanied by fatty infiltration and myatrophy. (*G*) Electron microscopy of subendocardial myocardial biopsy showing granular filamentous deposits within the cardiomyocyte cytoplasm. (*H*) Immunohistochemistry of right gastrocnemius muscle biopsy demonstrating desmin-positive aggregates (white arrows). (*I*) Electron microscopy of right gastrocnemius muscle biopsy showing electron-dense filamentous deposits with ultrastructural features similar to those observed in myocardial tissue.

Subendocardial myocardial biopsy revealed granular filamentous deposits within the myocardial cytoplasm (*Figure 1G*). Right gastrocnemius muscle biopsy demonstrated desmin-positive aggregates (*Figure 1H*) and electron-dense granular filament (*Figure 1I*) deposits with ultrastructural features similar to those in myocardial tissue, supporting the diagnosis of desmin myopathy with cardiomyopathy. Genetic testing identified a c.1360C > T mutation in the *DES* gene, resulting in a p.Arg454Trp substitution. This variant is pathogenic and responsible for desmin myopathy involving the myocardium.

Myopathies and cardiomyopathies related to myosin-associated proteins are rare inherited disorders. Integration of clinical manifestations, muscle enzyme evaluation, multimodal imaging, histopathology, and genetic testing improves diagnostic precision and allows dynamic assessment of disease progression and therapeutic response. Genetic disorders should be considered in patients presenting with ventricular conduction abnormalities, restrictive cardiomyopathy, and skeletal muscle atrophy.

## Data Availability

The data underlying this article cannot be shared publicly due to patient privacy concerns. The data are available from the corresponding author upon reasonable request.

## Notes

**Patient consent:** The authors confirm that written consent has been obtained from the patient for the submission and publication of this case report, including images and associated text, in accordance with the COPE guidelines.

